# Combination cyclin-dependent kinase 4/6 inhibitors and endocrine therapy versus endocrine monotherapy for hormonal receptor-positive, human epidermal growth factor receptor 2-negative advanced breast cancer: A systematic review and meta-analysis

**DOI:** 10.1371/journal.pone.0233571

**Published:** 2020-06-04

**Authors:** Jiani Zheng, Jingxun Wu, Chunyue Wang, Shiwen Zhuang, Jianbo Chen, Feng Ye

**Affiliations:** 1 First Clinical Medical School, Fujian Medical University, Fuzhou, China; 2 Department of Medical Oncology, Xiamen Cancer Hospital, The First Affiliated Hospital of Xiamen University, Xiamen, China; 3 Laboratory, Xiamen Cancer Hospital, The First Affiliated Hospital of Xiamen University, Xiamen, China; Fondazione IRCCS Istituto Nazionale dei Tumori, ITALY

## Abstract

**Purpose:**

This meta-analysis aimed to assess the efficacy and safety of cyclin-dependent kinase (CDK) 4/6 inhibitors plus endocrine therapy (ET) in hormonal receptor-positive (HR+), human epidermal growth factor receptor 2-negative (HER2-) advanced breast cancer (ABC).

**Methods:**

We searched PubMed, Embase, Cochrane, ClinicalTrials.gov., ASCO, ESMO and AACR databases from inception to October 10, 2019 for randomized controlled trials (RCTs) that compared CDK 4/6 inhibitors plus ET to single-agent ET with no treatment-line restriction. The main outcomes analyzed were progression-free survival (PFS), overall survival (OS), objective response rate (ORR), clinical benefit rate (CBR), and adverse events (AEs).

**Results:**

Of 938 identified studies, 9 RCTs with 5043 women were eligible and included. Compared with ET alone, CDK 4/6 inhibitors and ET combination improved in PFS (hazard ratio (HR) 0.54, 95% confidence interval (CI) 0.50–0.59, *p*< 0.00001) and OS (HR 0.77, 95% CI 0.69–0.85, *p*< 0.00001), regardless of ET strategies (HR 0.54, 95% CI 0.50–0.59 in PFS; HR 0.77, 95% CI 0.69–0.85 in OS), treatment line of advanced disease (HR 0.52, 95% CI 0.46–0.59 in PFS; HR 0.75, 95% CI 0.66–0.85 in OS) and menopausal status (HR 0.54, 95% CI 0.50–0.58 in PFS; HR 0.76, 95% CI 0.68–0.84 in OS). Higher risk of grade 3/4 AEs (RR 2.66, 95% CI 2.44–2.90, *p* < 0.00001) were observed in the combination group than in the ET group.

**Conclusions:**

Combination therapy with CDK 4/6 inhibitors and ET prolongs survival in HR+/ HER2- ABC. This combination is a better therapeutic strategy than endocrine monotherapy in HR+/HER2- ABC, regardless of treatment line, menopausal status and other individual characteristics.

## Introduction

As the most commonly diagnosed cancer among women, breast cancer is responsible for the highest cancer-related mortality [1]. Breast cancer has been characterized by the presence of multiple biomarkers. Hormone receptor-positive (HR+) and human epidermal growth factor receptor 2-negative (HER2-) constitutes 60%-65% of all the disease [2, 3]. Except for de novo disease is metastatic from the start, a proportion of patients with early breast cancer will progress to advanced disease during the treatment courses.

Endocrine therapy (ET) is the recommended first-line treatment regimen for HR+, HER2- ABC unless a visceral crisis or life-threating situation requires chemotherapy (CT) [4]. However, the intrinsic and acquired drug resistance, induced by the usage of single-agent ET, could induce progressive disease and/or late distant recurrence [5, 6]. Therefore, combination therapy strategies are being explored urgently to obstruct drug resistance and improve the long-term survival in HR+/HER2- ABC.

Cyclin-dependent kinases (CDKs) are a family of serine/threonine kinases that regulate the progression of the cell cycle. A number of preclinical experiments indicate that luminal breast cancer is hyperactive in CDK 4/6-cyclin D1, which provides great treatment efficacy to CDK 4/6 inhibitors [7, 8]. Impressive clinical efficacy in long-term disease control and progression-free survival (PFS) has been shown in clinical trials by adding CDK 4/6 inhibitors to endocrine therapy. Given the promising evidence in these trials, palbociclib, ribociclib and abemaciclib have been approved by the U.S. Food and Drug Administration (FDA) for the treatment of HR+ ABC [9].

However, several questions regarding combination treatment of these agents remain unclear. First, divergent treatment effects remain discovered between different clinical subgroups, especially the impact of race on PFS benefit [10]. Then, pooled analysis of the latest data of overall survival (OS) is still needed. Finally, adverse events (AEs), especially hematology toxicities between two arms (single-agent ET vs. combination therapy) need to be studied in a larger population in order to draw an objective conclusion. Therefore, this systematic review and meta-analysis of RCTs sought to establish the effects of CDK 4/6 inhibitors plus ET compared with single-agent ET on the key outcomes of PFS, OS, objective response rate (ORR), clinical benefit rate (CBR), and AEs.

## Methods

### Search strategy and selection criteria

This systematic review and meta-analysis are conducted and reported in accordance with the Preferred Reporting Items for Systematic Reviews and Meta-Analyses (PRISMA) Statement without protocol. We selected relevant studies published between Jan 1, 1990, and October 10, 2019, by searching PubMed, Embase, Cochrane and ClinicalTrials.gov. In addition, we searched the whole abstracts and meeting presentations from European Society for Medical Oncology (ESMO), American Society of Clinical Oncology (ASCO) and American Association for Cancer Research (AACR). We also conducted a manual search of the reference lists of key articles.

The following combined text and MeSH terms: “breast cancer” and “cyclin dependent kinases”, but deleted ‘endocrine therapy (MeSH)’ in the search terms due to the expansion of too many irrelevant studies. The complete search used for PubMed was: (Breast Neoplasms [MeSH Terms] OR breast cancer* [Title/Abstract] OR breast carcinom* [Title/Abstract] OR breast tumour* [Title/Abstract] OR breast malignan* [Title/Abstract]) AND (Cyclin-Dependent kinases [MeSH Terms] OR cyclin-dependent kinase inhibitor* [Title/Abstract] OR cyclin D-cyclin-dependent kinase inhibitor* [Title/Abstract] OR CDK 4/6 inhibitor* [Title/Abstract] OR cyclin-dependent kinase 4/6 inhibitor* [Title/Abstract] OR palbociclib [Title/Abstract] OR ribociclib [Title/Abstract] OR abemaciclib [Title/Abstract]) AND (randomized controlled trial [Publication Type] OR controlled clinical trial [Publication Type]).

### Study selection

Inclusion criteria were as follows: (1) phase II or III randomized clinical trials; (2) eligible adults with HR+, HER2- advanced breast cancer, compared combination treatment of CDK 4/6 inhibitors and endocrine therapy to single-agent endocrine therapy; (4) The trials reported with enough data for the pooled analysis.

Exclusion criteria were as follows: (1) retrospective and observational studies; preclinical trials, phase I clinical trials and non-randomized trials studies; (2) CDK4/6 inhibitors for adjuvant or neoadjuvant therapy in early-stage breast cancer; (3) duplicates of previous publications.

### Data extraction and quality assessment

The databases were searched by two investigators (ZJN and WJX) independently. Then, the following data were extracted from the selected studies: trial name, publication year, trial phase, number of participants, age, histology, treatment strategy, treatment regimen and dose, median follow-up, ORR, median PFS and median OS. The outcomes assessed were as follows: hazard ratio (HR) with 95% confidential interval (CI) for PFS and OS; number of patients who experienced a partial response or complete response as ORR; number of patients who experienced a stable disease, partial response or complete response as CBR; number of patients that developed grade 3/4 AEs.

Two independent reviewers (ZJN and WJX) assessed risk for bias according to the Cochrane Collaboration and the PRISMA recommendations. The disagreements were discussed and resolved by consensus.

### Statistical analysis

All statistical analyses were performed using Review Manager ver.5.3 software and STATA ver.15.0 software in accordance with Cochrane Collaboration guidelines for Meta-analysis. The survival outcomes such as PFS and OS were calculated as hazard ratio (HR). Dichotomous variables such as ORR, CBR and AEs were calculated as relative risk (RR). The χ^2^-test and I^2^ statistics were used to evaluate statistical heterogeneity. The heterogeneity was regarded as substantial if the I^2^ value was greater than 30% or a low *p* -value (< 0.10) was found in the Chi^2^ test. The pooled results of each study were calculated by fixed-effects (Mantel–Haenszel method) model, and a random-effects model (DerSimonian-Laird method) was applied if moderate heterogeneity (I^2^> 30% or *p* -value< 0.10) was found. A p < 0.05 was established as statistical significance. Since several RCTs reported HR separately for ‘Caucasian and other’/‘white and black’, we combined these two groups into a single group as ‘non-Asians’ and combined hazard ratio using a fixed-effects model to discern PFS differences between race, as previously described [11].

A sensitivity study was used to identify any individual study that significantly influenced the overall estimates by excluding each study repeatedly and calculating the pooled estimates for the remaining studies.

## Results

### Characteristics of the eligible studies

**Fig 1:**
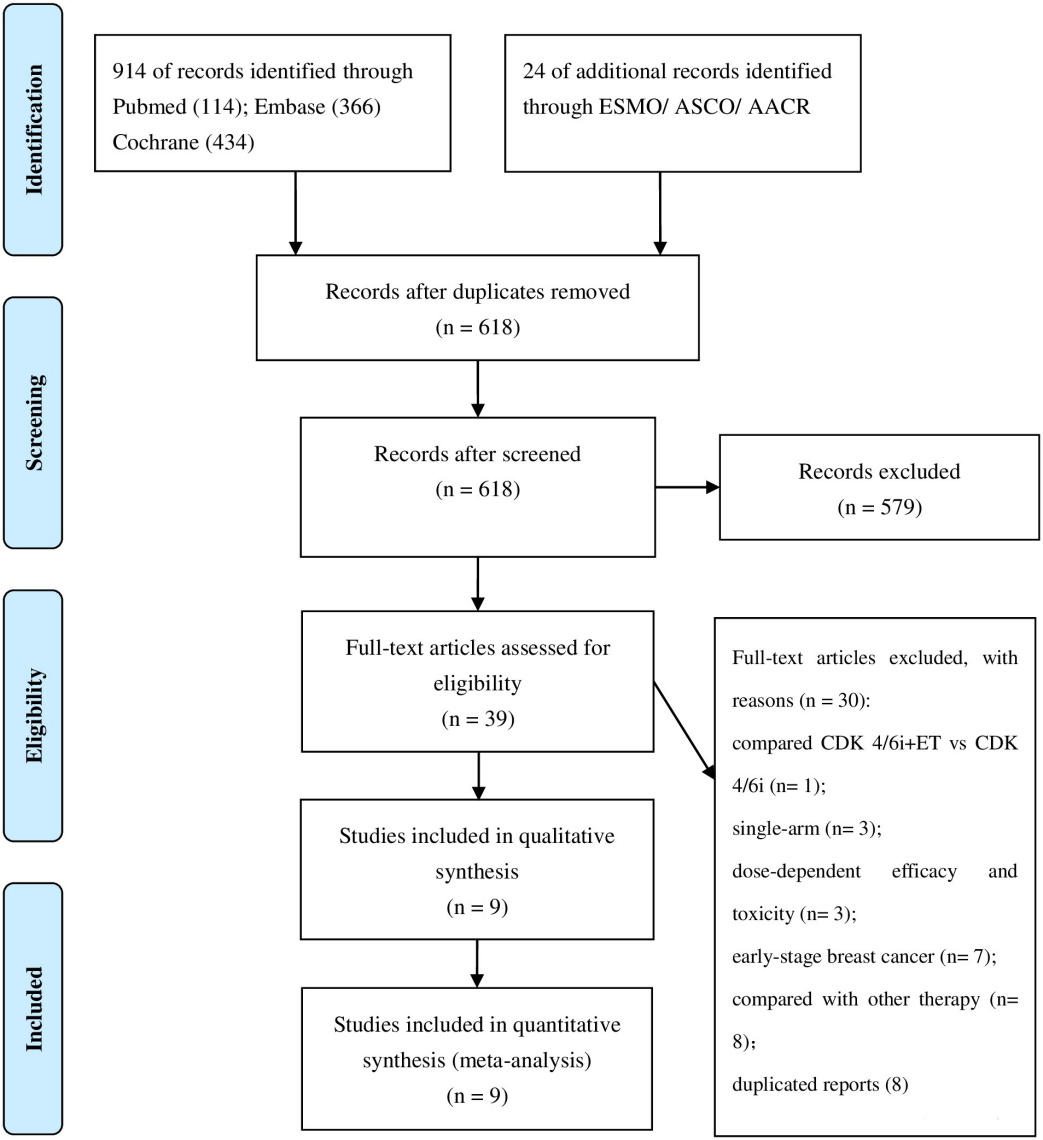
Flow diagram for inclusion and exclusion of studies. ESMO: European Society for Medical Oncology; ASCO: American Society of Clinical Oncology; AACR: American Association for Cancer Research.

A total of 9 eligible studies (N = 5043) were included in this analysis (Fig 1). Among the 9 enrolled studies, 3 were palbociclib trials [12–14], 3 were riboliclib trials [15–17] and 3 were abemaciclib trials [18, 19]. As for menopausal status, 6 trials included treatment of postmenopausal women [12, 13, 15, 16, 19, 20], 1 trial treated pre- or perimenopausal women [17] and 2 trials treated women with both menopausal status [14, 18]. As for combination schemes, 5 trials applied first-line ET strategy in our studies [12, 13, 15, 17, 19], and 4 trials included first-line and subsequent-line ET strategies simultaneously [14, 16, 18, 20]. More detailed characteristics of the 9 studies are presented in Table 1. The ORR, median PFS, OS, and the reported HR, 95% CI, *p* -value were extracted from the published trials are presented in Table 2.

**Table 1:** Characteristic of nine included trials.

Study	Year	Phase	Histology	region	Regimen	Dose	Patients	Median age (year)	Treatment strategy for ABC
**PALOMA-1** **NCT00721409**	2017	II	Postmenopausal women; ER+/HER2-ABC	International	Palbociclib+ Letrozole vs Letrozole	Palbociclib 125mg daily, 3 weeks on/ 1 week off; LTZ 2.5mg qd	165 (84/81)	63 64	First-line therapy
**PALOMA-2** **NCT01740427**	2018	III	Postmenopausal women; ER+/HER2-ABC	International	Palbociclib+ Letrozole vs Placebo+ Letrozole	Palbociclib 125mg daily, 3 weeks on/ 1 week off; LTZ 2.5mg qd	666 (444/222)	62 61	First-line therapy
**PALOMA-3** **NCT01942135**	2018	III	Women; HR+/HER2- ABC	International	Palbociclib+ Fulvestrant vs Placebo+ Fulvestrant	Palbociclib 125mg daily 3 weeks on/ 1 week off; Fulvestrant 500mg q4w (additional on d15 of cycle 1)	521 (347/174)	57 56	First-line or Subsequent-line ET; ≤1 line CT
**MONALEESA-2** **NCT01958021**	2019	III	Postmenopausal women; HR+/HER2-ABC	International	Ribociclib+ Letrozole vs Placebo+ Letrozole	Ribociclib 600mg daily 3 weeks on/ 1 week off; LTZ 2.5mg qd	668 (334/334)	62 63	First-line therapy
**MONALEESA-3** **NCT02422615**	2018	III	Postmenopausal women; HR+/HER2-ABC	International	Ribociclib+ Fulvestrant vs Placebo+ Fulvestrant	Ribociclib 600mg daily 3 weeks on/ 1 week off; Fulvestrant 500mg q4w (additional on d15 of cycle 1)	726 (484/242)	63 63	First-line or Second-line ET; no CT
**MONALEESA-7** **NCT02278120**	2019	III	Pre- or peri-menopausal Women; HR+/HER2- ABC	International	Ribociclib+ TAM/NSAI + Goserelin vs Placebo + TAM/NSAI + Goserelin	Ribociclib 600mg daily 3 weeks on/ 1 week off; 20mg qd; TAM 20mg qd OR LTZ 2.5mg qd OR Anastrozole 1mg qd; Goserelin 3.6mg q4w	672 (335/337)	43 45	First-line ET; ≤1 line CT
**MONARCH-2** **NCT02107703**	2019	III	Women; HR+/HER2- ABC	International	Abemaciclib+ Fulvestrant vs Placebo+ Fulvestrant	Abemaciclib 150mg bid; Fulvestrant 500mg q4w (additional on d15 of cycle 1)	669 (446/223)	59 62	First-line or Second-line ET; no CT
**MONARCH-3** **NCT02246621**	2019	III	Postmenopausal women; HR+/HER2-ABC	International	Abemaciclib+ NSAI vs Placebo+ NSAI	Abemaciclib 150mg bid; LTZ 2.5mg qd OR Anastrozole 1mg qd;	493 (328/165)	63 63	First-line therapy
**MONARCH plus** **NCT02763566**	2019	III	Postmenopausal women; HR+/HER2- ABC	International	Abemaciclib+ NSAI vs Placebo+ NSAI Abemaciclib+ Fulvestrant vs Placebo+ Fulvestrant	Abemaciclib 150mg bid; LTZ 2.5mg qd OR Anastrozole 1mg qd; Fulvestrant 500mg q4w (additional on d15 of cycle 1)	463 (207/99) (104/53)	- -	First-line therapy/ subsequent-line ET ≤1 line CT

ER+: Estrogen receptor positive; HR+: Hormonal receptor-positive; HER2-: Human epidermal growth factor receptor 2-negative; ABC: Advanced breast cancer; NSAI: Nonsteroidal aromatase inhibitor (letrozole or anastrozole); ET: endocrine therapy; CT: chemotherapy; LTZ: Letrozole; TAM: tamoxifen; NR: Not reached.

**Table 2:** Medium follow-up, objective response rate, medium progression-free survival and overall survival of included trials.

Study	Regimen	Patients	Median follow-up	ORR (%)	Median PFS			OS		
					Months	Reported HR	P-value	Months	Reported HR	P-value
			Months (95% CI)		(95% CI)	(95% CI)		(95% CI)	(95% CI)	
**PALOMA-1** **NCT00721409**	Palbociclib+ Letrozole vs Letrozole	84	>29.6(27.9–36.0)	42.9	20.2(13.8–27.5)	0.488(0.319–0.748)	0.0004	37.5(31.4–47.8)	0.897(0.623–1.294)	0.281
		81	>27.9(25.5–31.1)	33.3	10.2(5.7–12.6)			34.5(27.4–42.6)		
**PALOMA-2** **NCT01740427**	Palbociclib+ Letrozole vs Placebo+ Letrozole	444	37.6(37.2–38.0)	42.1	27.6(22.4–30.3)	0.563(0.461–0.687)	<0.0001	NR		
		222	37.3(36.3–37.9)	34.7	14.5(12.3–17.1)					
**PALOMA-3** **NCT01942135**	Palbociclib+ Fulvestrant vs Placebo+ Fulvestrant	347	44.8	19	11.2(9.5–12.9)	0.50(0.40–0.62)	0.0001	34.9(28.8–40.0)	0.81(0.64–1.03)	0.09
		174		9	4.6(3.5–5.6)			28.0(23.6–34.6)		
**MONALEESA-2** **NCT01958021**	Ribociclib+ Letrozole vs Placebo+ Letrozole	334	39.4	42.5	25.3(23.0–30.3)	0.568(0.457–0.704)	<0.0001	NR(NR-NR)	0.746(0.517–1.078)	NE
		334		28.7	16.0(13.4–18.2)			33.0(33.0-NR)		
**MONALEESA-3** **NCT02422615**	Ribociclib+ Fulvestrant vs Placebo+ Fulvestrant	484	39.4	32.4	20.5(18.5–23.5)	0.593(0.480–0.732)	<0.01	NR	0.724(0.568–0.924)	0.00455
		242		21.5	12.8(10.9–16.3)			40.0		
**MONALEESA-7** **NCT02278120**	Ribociclib+ TAM/NSAI+ Goserelin vs Placebo+ TAM/NSAI+ Goserelin	335	34.6	41	23.8(19.2-NR)	0.55(0.44–0.69)	<0.0001	NR	0.71(0.54–0.95)	0.00973
		337		30	13.0(11.0–16.4)			40.9(37.8-NE)		
**MONARCH-2** **NCT02107703**	Abemaciclib+ Fulvestrant vs Placebo+ Fulvestrant	446	47.7	35.2	16.4	0.553(0.449–0.681)	<0.01	46.7	0.757(0.606–0.945)	0.01
		223		16.1	9.3			37.3		
**MONARCH-3** **NCT02246621**	Abemaciclib+ NSAI vs Placebo+ NSAI	328	26.7	49.7	28.18	0.540(0.418–0.698)	0.00002	NR		
		165		37.0	14.76					
**MONARCH plus (cohort A)** **NCT02763566**	Abemaciclib+ NSAI vs Placebo+ NSAI	207	-	56.0	NR	0.499(0.346–0.719)	0.0001	NR		
		99		30.3	14.73					
**MONARCH plus (cohort B)** **NCT02763566**	Abemaciclib+ Fulvestrant vs Placebo+ Fulvestrant	104	-	38.5	11.47	0.376(0.240–0.588)	<0.0001	NR		
		53		7.5	5.59					

ORR: objective response rate; PFS: progression free survival; OS: overall survival; HR: hazard ratio; CI: confidence interval; NSAI: Nonsteroidal aromatase inhibitor (letrozole or anastrozole); TAM: tamoxifen; NR: Not reached; NE: the value could not be estimated.

### Quality of studies

**Fig 2:**
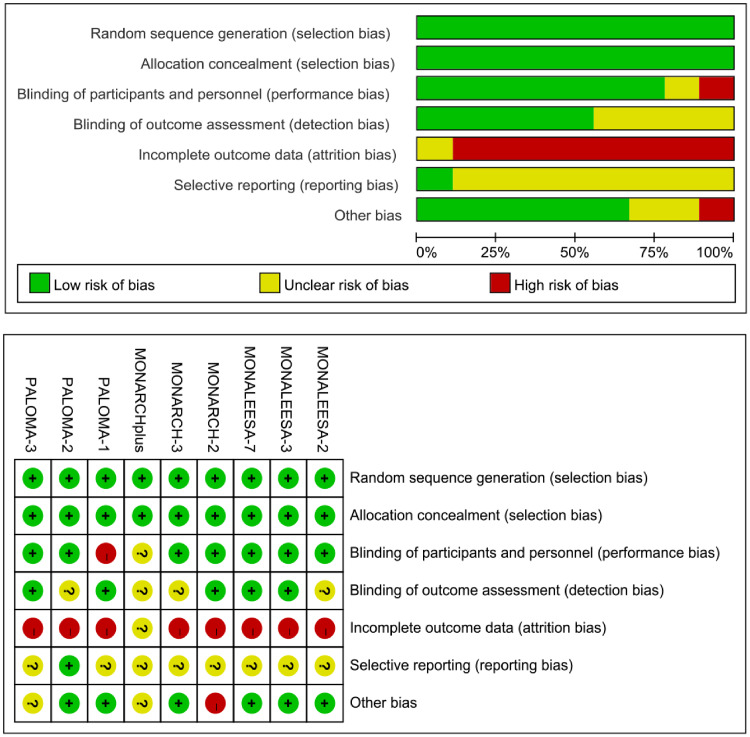
Risk of bias for selected studies.

According to the published articles or posted final protocol, all trials were at low risk of selection bias (random sequence generation and allocation concealment). Except for one trial was open-label trials, other RCTs were double-blind trials with low risk of performance bias. Most of the included randomized trials had a low risk of detection bias, reporting bias, and other bias. Eight of nine trials were at high risk of attrition bias because of more than 50% discontinued patients after randomization and receiving at least one dose of allocated intervention. However, objective progression or relapse caused approximately 50–80% of patients withdrew from the included RCTs. Such patients received other subsequent treatment with continuous follow-up. Therefore, the included studies had a low risk of incomplete outcome data. Indeed, the high attrition bias in the present study did not influence the result of this meta-analysis (Fig 2).

### PFS

**Fig 3:**
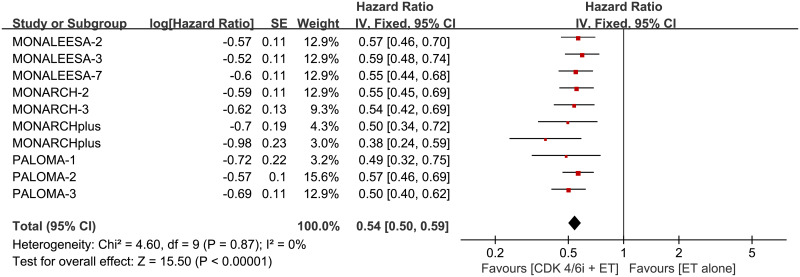
Forest plot of pooled hazard ratio for progression-free survival (PFS) in CDK 4/6 inhibitors plus endocrine combination therapy and endocrine monotherapy. SE: standard error; CDK 4/6i: CDK 4/6 inhibitors; ET: endocrine therapy.

Prolongation of PFS were achieved by adding CDK 4/6 inhibitors to endocrine therapy in individual RCTs (Table 2). The pooled HR showed a significant improvement in PFS for combination therapy over ET alone (Fig 3; HR 0.54, 95% CI 0.50–0.59, *p* < 0.00001, I^2^ for heterogeneity = 0%, *p* = 0.88).

#### Subgroup analysis of PFS

**Fig 4:**
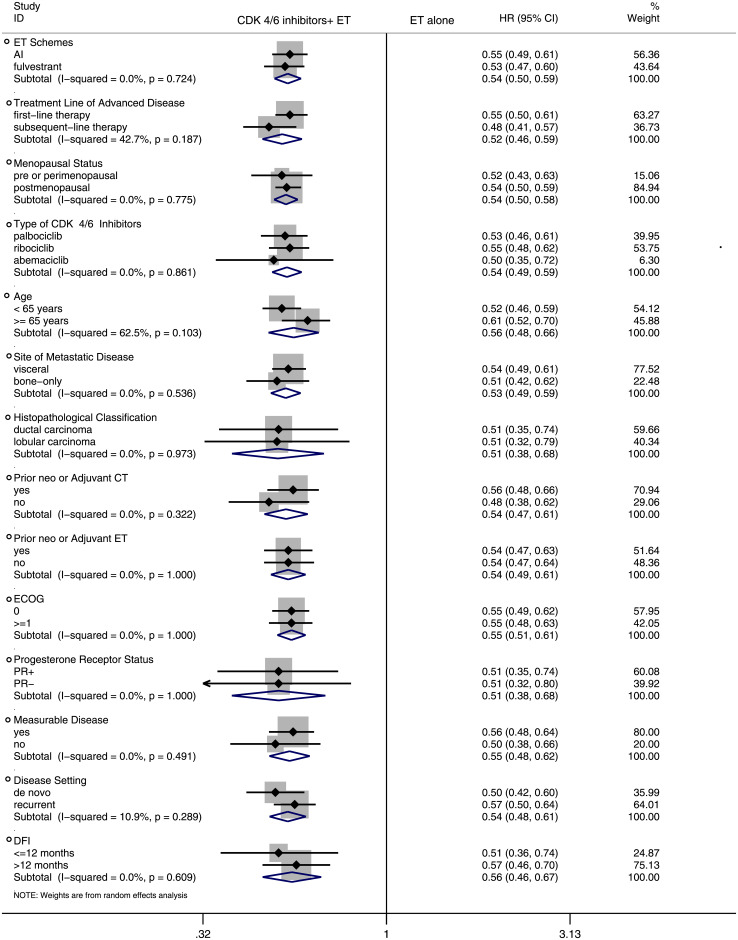
Forest plot of hazard ratio for progression-free survival (PFS) by subgroup analysis in CDK 4/6 inhibitors plus endocrine combination therapy and endocrine monotherapy. ET: endocrine therapy; AI: aromatase inhibitors; CT: chemotherapy; PR: progesterone receptor; DFI: disease-free interval.

**Fig 5:**
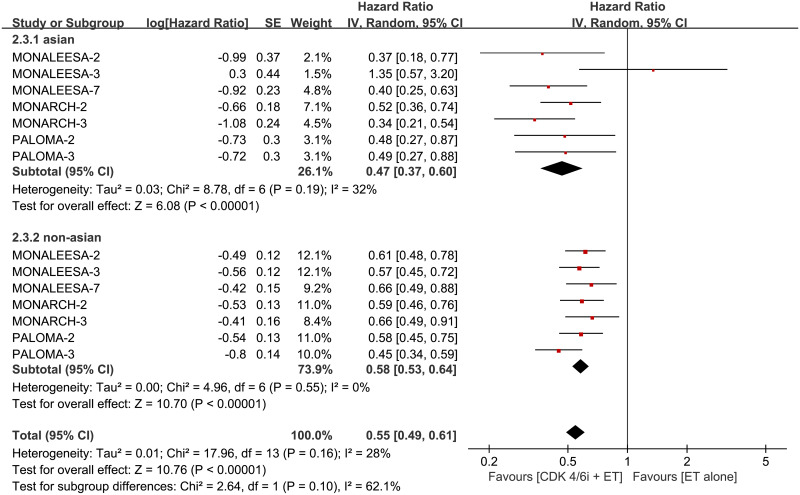
Forest plot of hazard ratio for progression-free survival (PFS) for Asian and non-Asian subgroups in CDK 4/6 inhibitors plus endocrine combination therapy and endocrine monotherapy.

To assess whether PFS varied across clinical subgroups, the included studies were subgrouped as: “ET schemes”, “treatment line of advanced disease”, “menopausal status”, “type of CDK4/6 inhibitors”, “age”, “site of metastatic disease”, “histopathological classification”, “prior neoadjuvant or adjuvant CT”, “prior neoadjuvant or adjuvant ET”, “ECOG”, “progesterone receptor status”, “measurable disease”, “disease setting”, “disease-free interval (DFI)” (Fig 4) and “race” (Fig 5). The original analysis figures were collected in S1-S10 Fig in S1 File. The analysis demonstrated consistent treatment effects across the majority of subgroups.

### OS

**Fig 6:**
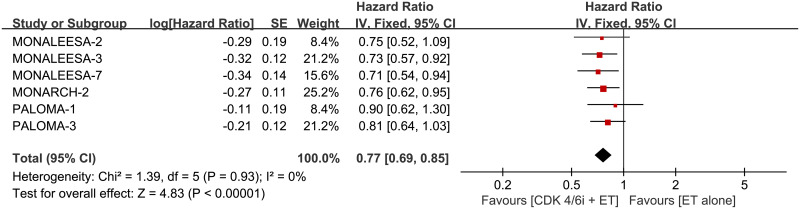
Forest plot of pooled hazard ratio for overall survival (OS) in CDK 4/6 inhibitors plus endocrine combination therapy and endocrine monotherapy.

Combination therapy also increased the OS compared with single-agent ET (Fig 6; HR 0.77, 95% CI 0.69–0.85, *p* < 0.00001, I^2^ for heterogeneity = 0%, *p* = 0.93).

#### Subgroup analysis of OS

**Fig 7:**
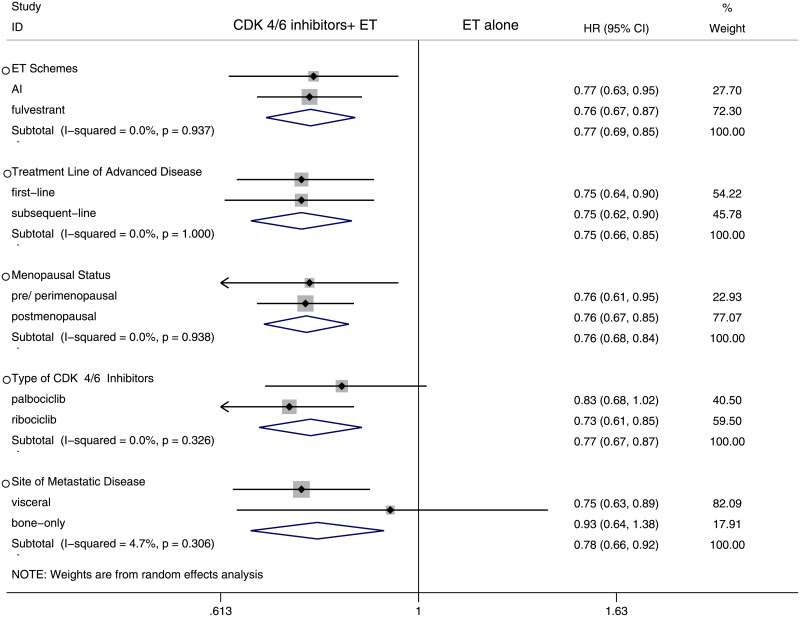
Forest plot of hazard ratio for overall survival (OS) by subgroup analysis in CDK 4/6 inhibitors plus endocrine combination therapy and endocrine monotherapy.

No statistically differences were shown within the stratification factor of ET schemes, treatment line of advanced disease and menopausal status (Fig 7). The original analysis figures were collected in S11 Fig in S1 File.

In the analysis of type of CDK 4/6 inhibitors, compared with ribociclib (HR 0.73, 95% CI 0.61–0.85), palbociclib was not observed to have an OS benefit from combination therapy (HR 0.83, 95% CI 0.68–1.02). Moreover, bone-only metastatic showed no statistically benefit from combination therapy (HR 0.93, 95% CI 0.64–1.38). In contrast, improved OS was observed in patients with visceral metastatic (HR 0.75, 95% CI 0.63–0.89) (Fig 7).

**Fig 8:**
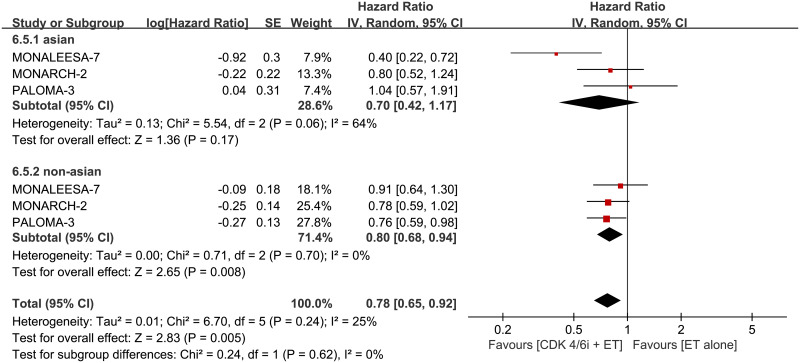
Forest plot of hazard ratio for overall survival (OS) for Asian and non-Asian subgroups in CDK 4/6 inhibitors plus endocrine combination therapy and endocrine monotherapy.

Different from the impact of race on PFS, combination therapy did not improve OS in Asians (HR 0.70, 95% CI 0.42–1.17, *p* = 0.17, I^2^ for heterogeneity = 64%, *p* = 0.06) compared to non-Asians (HR 0.80, 95% CI 0.68–0.94, *p* = 0.008, I^2^ for heterogeneity = 0%, *p* = 0.70) (Fig 8).

### ORR and CBR

**Fig 9:**
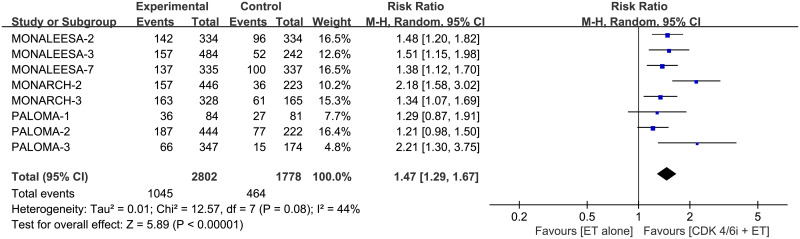
Forest plot of pooled relative risk for objective response rate (ORR) in CDK 4/6 inhibitors plus endocrine combination therapy and endocrine monotherapy.

**Fig 10:**
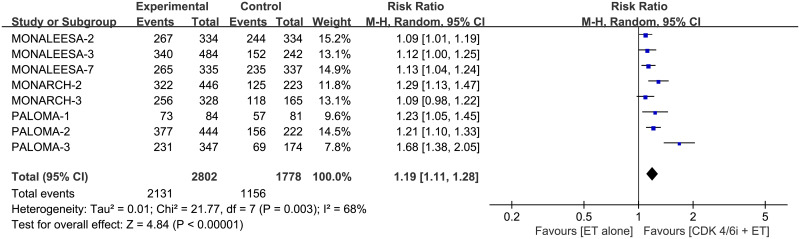
Forest plot of pooled relative risk for clinical benefit rate (CBR) in CDK 4/6 inhibitors plus endocrine combination therapy and endocrine monotherapy.

The pooled HR showed that combination therapy improves both ORR (RR 1.47, 95% CI 1.29–1.67, *p* < 0.00001, I^2^ for heterogeneity = 44%, *p* = 0.08) and CBR (RR 1.19, 95% CI 1.11–1.28, *p* < 0.00001, I^2^ for heterogeneity = 68%, *p* = 0.003) than monotherapy (Figs 9 and 10).

### Adverse events

**Fig 11:**
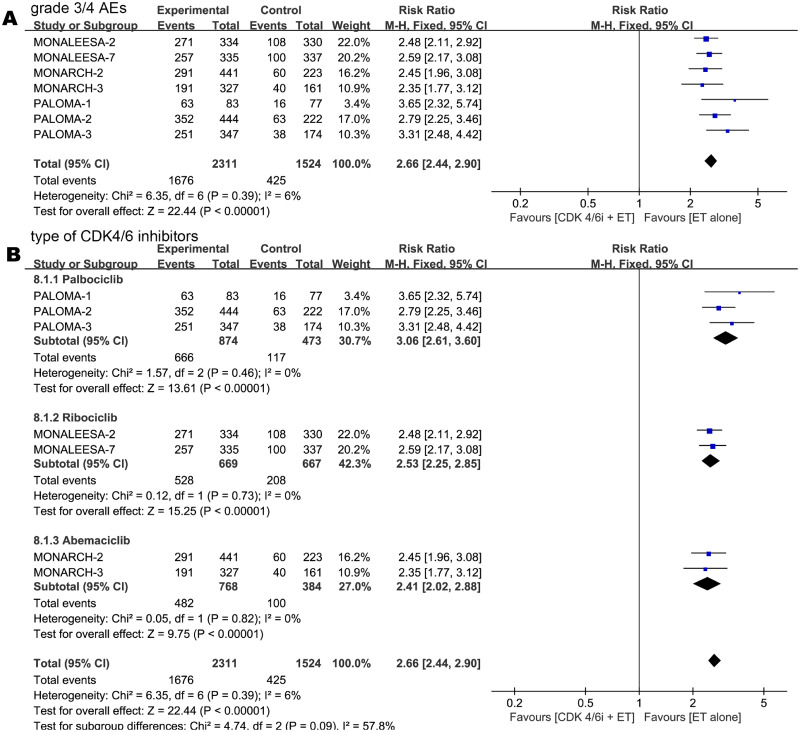
Forest plot of pooled relative risk for grade 3/4 adverse events (AEs) in CDK 4/6 inhibitors plus endocrine combination therapy and endocrine monotherapy (A). Relative risk for grade 3/4 adverse events (AEs) by subgroup in type of CDK inhibitors between two treatment groups (B).

Except for MONALEESA-3, all trials included in this meta-analysis reported the total number of grade 3/4 treatment-related AEs. In the combination arm, the incidence of grade 3/4 AEs was significantly increased compared to that in the single-agent arm (RR 2.66, 95% CI 2.44–2.90, *p* < 0.00001, I^2^ for heterogeneity = 6%, *p* = 0.39) (Fig 11A).

#### Subgroup analysis of AEs

No statistically difference were found between palbociclib (RR 3.06, 95% CI 2.61–3.60, *p* < 0.00001, I^**2**^ for heterogeneity = 0%, *p* = 0.46), ribociclib (RR 2.53, 95% CI 2.25–2.85, *p* < 0.00001, I^**2**^ for heterogeneity = 0%, *p* = 0.73) and abemaciclib (RR 2.41, 95% CI 2.02–2.88, *p* < 0.00001, I^**2**^ for heterogeneity = 0%, *p* = 0.82) in grade 3/4 AEs between combination therapy and single-agent therapy (Fig 11B).

**Fig 12:**
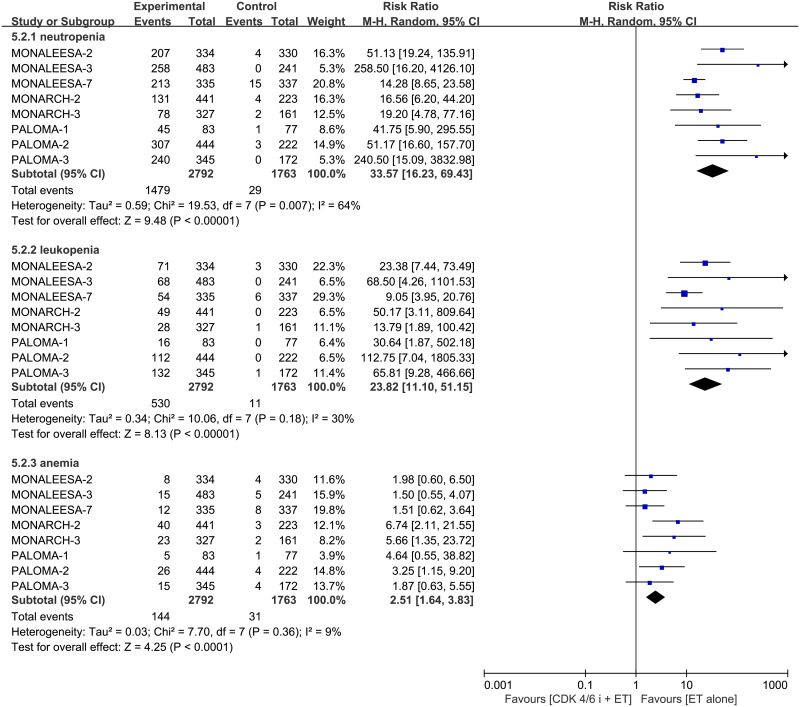
Forest plot of pooled relative risk for hematology toxicities (neutropenia, leukopenia, anemia) in CDK 4/6 inhibitors plus endocrine combination therapy and endocrine monotherapy.

Analyzed of three of the most common hematology adverse events, neutropenia, leukopenia and anemia, we found that CDK 4/6 inhibitors plus ET significantly increased the incidence of neutropenia (RR 33.57, 95% CI 16.23–69.43, *p* < 0.00001, I^2^ for heterogeneity = 64%, *p* = 0.007), leukopenia (RR 23.82, 95%CI 11.10–51.15, *p* < 0.00001, I^2^ for heterogeneity = 30%, *p* = 0.18) and anemia (RR 2.51, 95% CI 1.64–3.83, *p* < 0.0001, I^2^ for heterogeneity = 9%, *p* = 0.36) compared to single-agent ET (Fig 12).

### Sensitivity analysis

We re-analyzed the data by omitting individual trials. The corresponding pooled RRs and HRs were not qualitatively altered in sensitivity analysis.

In terms of the subgroup of Asian and non-Asian in PFS, the pooled HR was not significantly changed during excluded each trial. However, the p-value of test for subgroup differences changed from 0.10 to 0.01 once excluded MONALEESA-3 (S12 Fig in S1 File). At the same time, I^2^ for heterogeneity reduced from 28% to 13%. Importantly, such sensitivity analysis showed the PFS benefit difference may exist in ethnicity.

## Discussion

As one of the combination schemes with endocrine therapy, CDK 4/6 inhibitors are administered as first-line or subsequent-line therapy for ABC in clinical trials. The National Comprehensive Cancer Network (NCCN) has already recommended CDK 4/6 inhibitors plus ET for postmenopausal ABC with no prior endocrine therapy within one year [9]. However, according to the latest data, the effect of such a combination strategy is not limited to the first-line treatment and postmenopausal patients [14, 16–18]. In the present study, we analyzed the efficacy and toxicity of CDK 4/6 inhibitors plus endocrine therapy compared to that of endocrine monotherapy, and further aimed to identify the potential candidates most likely to respond to combination therapy.

A similar meta-analysis included seven RCTs for HR+/HER2- advanced breast cancer [21], but the analyzed data were only collected until March 2018. Recently, six RCTs provided updated data and two RCTs initially posted the results [16, 20, 22–29]. Using the latest data, we enhanced the results of the previous meta-analysis in overall survival (OS) and clinical subgroups of survival data.

Results of the present study lend support to the survival benefits of CDK 4/6 inhibitors and ET combination treatment. This analysis indicated that PFS in patients undergoing combination therapy is superior to endocrine monotherapy, which is consistent with the previous meta-analysis [21]. The HR and 95% CI in our study (HR 0.54, 95% CI 0.50–0.59, *p* < 0.00001) were similar to those in the study of Deng et al. (HR 0.54, 95% CI 0.49–0.59) even though we newly added two RCTs and updated four RCTs. Furthermore, we initially included six RCTs to analyze the OS between the two treatments. The median follow-up time of these included trials were more than 34.6 months. The statistical advantage improvement of OS suggests that benefit seen in PFS will likely translate to a prolongation of OS. Except for PALOMA-1 and PALOMA-3, half of the included RCTs increased OS through administration of combination therapy. Particularly, MONALEESA-3 and MONALEESA-7 both show a consistently and statistically prolongation of survival [30]. As an open-label trial, PALOMA-1 might have induced performance bias. In addition, PALOMA-1 required ER+/ HER2- ABC patients in both 1 and 2 cohorts. Patients in cohort 2 were additionally required to contain diseases with amplification of cyclin D1 (CCND1), loss of p16 (INK4A or CDKN2A), or both. After realizing that two eligible cohorts were not different in outcomes, PALOMA-1 amended the statistical analysis such that combined analysis the primary endpoint in two cohorts may also explain the failure to increase the OS. Then, the OS results did not meet the prespecified threshold for statistical significance, but PALOMA-3 resulted in an absolute prolongation of patient’ OS of 6.9 months.

Excluded MONALEESA-3 which only has 22 and 7 Asian events in experimental and control arms for PFS analysis, there was a significant interaction between PFS and race (*p* = 0.01). Our analysis showed that Asians can benefit from combination treatment in PFS but not in OS, but non-Asians displayed an improvement both in PFS and OS. A meta-analysis published in 2018 demonstrated that the magnitude of PFS benefit is race-dependent. There was a significant interaction between PFS and race (*p* = 0.002) [31], and the result of the present study verified this view. Finally, the authors stated a hypothesis that interethnic differences in drug exposure and genetic polymorphisms of CYP3A4 between different race may explain the above finding. Lacking data of OS, the efficacy discussion of the previous meta-analysis limited in PFS. Our data showed that PALOMA-3 and MONARCH-2 which included first-line and subsequent-line ET strategies simultaneously, showed OS benefits in non-Asian but not in Asian. A similar finding was reported for MONALEESA-3, another first-line and subsequent-line study [30]. Only included first-line ET strategies, MONALEESA-7 showed that combination therapy improved OS in Asian instead of non-Asian. Possibly due to the small Asian sample size, the race subgroup of OS seemed variability. The ongoing MONARCH-plus were predominantly including Chinese patients and wPATHWAY (NCT03423199) activates in the Asian region will help to investigate the efficacy and toxicity of CDK 4/6 inhibitors in Asian. Furthermore, the final second analysis of existing RCTs even head-to-head RCTs are looking forward to answering such conflicting findings.

Similarly, in patients with visceral metastatic at baseline, which is noted as a poor prognostic subgroup, more significantly OS improvement was found than in a better prognostic subgroup of bone-only disease [29]. Since visceral metastatic appears worse malignant biological behavior, earlier separation of Kaplan-Meier survival curves and a larger effect were showed in all included studies. Actually, the total number of deaths/ total patients were 382/ 1026 and 113/339 in visceral metastatic and bone-only disease group, respectively. Such inadequate sample size of bone -only caused 17.91% weight versus 82.09% weight in visceral metastatic.

The preclinical trials indicated that palbociclib and ribociclib have a similar chemical structure, while abemaciclib presents a higher selectivity for the complex CDK 4/cyclin D1 [32]. However, the difference of chemical structure do not appears in the analysis. The PFS was similar between the three arms of that study. As for the divergence in OS was showed between palbociclib and ribociclib, the different experimental designs of PALOMA-1 and PALOMA-3 contextually may have induced the negative result. Therefore, additional follow-up of OS subgroups remain to be analyzed further.

Overall, this study has shown that combination therapy significantly improves PFS and OS regardless of the differences between AI or fulvestrant, first-line or subsequent-line for advanced disease and pre/perimenopausal or postmenopausal. Also, there were no obvious PFS differences between the study arms in age, site of metastatic disease, histopathological classification, prior neoadjuvant or adjuvant CT, prior neoadjuvant or adjuvant ET, ECOG, progesterone receptor status, measurable disease, disease setting and DFI.

The clinically meaningful and statistically significant ORR and CBR benefits were observed in the combination treatment arm. Three RCTs included first-line and subsequent-line simultaneously. The proportion of subsequent-line treatment was more than 75% in PALOMA-3 compared with nearly 59% in MONALEESA-3 and MONARCH-2, which resulted in heterogeneity among the analyses of ORR and CBR. Notably, the sensitivity analysis showed the influence of bias among PALOMA-3 in this study.

Meanwhile, a significantly higher risk of major grade 3/4 hematologic toxicities (neutropenia, leukopenia and anemia) were observed in our analysis, consistent with the on-target inhibition of CDKs 4 and 6, which are highly expressed in hematopoietic stem cells [33]. A recent meta-analysis considered that palbociclib and ribociclib had a similar rate of grade 3/4 AEs while abemaciclib had a lower rate of grade 3/4 AEs overall [34]. However, total grade 3/4 AEs were no obviously different between varied types of CDK4/6 inhibitors in the present study (*p* = 0.09).

A limitation of this analysis is that the interim analysis of OS was still immature in several trials. Although the pooled analysis was statistically definitive, the included data were insufficient for subgroup analysis. Second, all of included RCTs were possible to carry the potential risk of bias such as open-label in one RCT and funding in all RCTs. Third, differences exist in eligibility criteria, such as whether prior chemotherapy for advanced disease was allowed, which result in diversity between ‘first-line/subsequent-line treatment’ and ‘first-line/subsequent-line endocrine treatment’. Due to the fact that three in four RCTs were short of the PFS data in endocrine treatment line for ABC, we only selected ‘first-line/subsequent-line treatment’ for further subgroup analysis. Additionally, compared with aromatase inhibitors only for first-line treatment, fulvestrant was given as first and subsequent-line in the included studies. In two studies (FALCON and FIRST) [35, 36], fulvestrant is superior to anastrozole for patients with HR+/HER2- ABC. Therefore, head-to-head studies are needed to establish the combination therapy of fulvestrant and CDK4/6 inhibitors as first-line treatment in HR+ ABC.

Results of this meta-analysis show that, compared with endocrine monotherapy, combination treatment with CDK 4/6 inhibitors and ET can yield improved PFS and OS. Furthermore, compared with ET alone, this combination offers greater ORR and CBR, but also increases total grade 3/4 AEs and hematologic-specific toxicities. These data thus lend support to CDK 4/6 inhibitors and ET combination treatment as first-line and subsequent-line treatment in patients with HR+, HER2- advanced breast cancer, without the limitation of patients’ or disease characteristics.

## Supporting information

S1 File (PDF)

S1 Data (DOC)
