# Supplementary material for: Combination cyclin-dependent kinase 4/6 inhibitors and endocrine therapy versus endocrine monotherapy for hormonal receptor-positive, human epidermal growth factor receptor 2-negative advanced breast cancer: A systematic review and meta-analysis
Source: PLoS One. 2020 Jun 4;15(6):e0233571. doi: 10.1371/journal.pone.0233571 (PMC7272037; doi:10.1371/journal.pone.0233571)
Supplement: S1 File — (PDF) [file pone.0233571.s001.pdf]

Title: Combination cyclin-dependent kinase 4/6 inhibitors and endocrine therapy versus endocrine monotherapy for hormonal receptor-positive, human epidermal growth factor receptor 2-negative advanced breast cancer: A systematic review and meta-analysis

Jiani Zheng[1,2,3*], Jingxun Wu[1,2,3*], Chunyue Wang [1,2,3], Shiwen Zhuang [1,2,3], Jianbo Chen[1,2,3], Feng Ye[1,2,3]

1. First Clinical Medical School, Fujian Medical University, Fuzhou, China;

2. Department of Medical Oncology, Xiamen Cancer Hospital, The First Affiliated Hospital of Xiamen University, Xiamen, China;

3. Laboratory, Xiamen Cancer Hospital, The First Affiliated Hospital of Xiamen University, Xiamen, China;

Corresponding author: Feng Ye

Department of Medical Oncology, Xiamen Cancer Hospital, The First Affiliated Hospital of Xiamen University, 55 Zhenhai Road, Xiamen, Fujian 361000, China; Tel: +86-592-2139634; Fax: +86-592-2139571; Email: yefengdoctor@xmu.edu.cn

* These authors contributed equally to this paper.

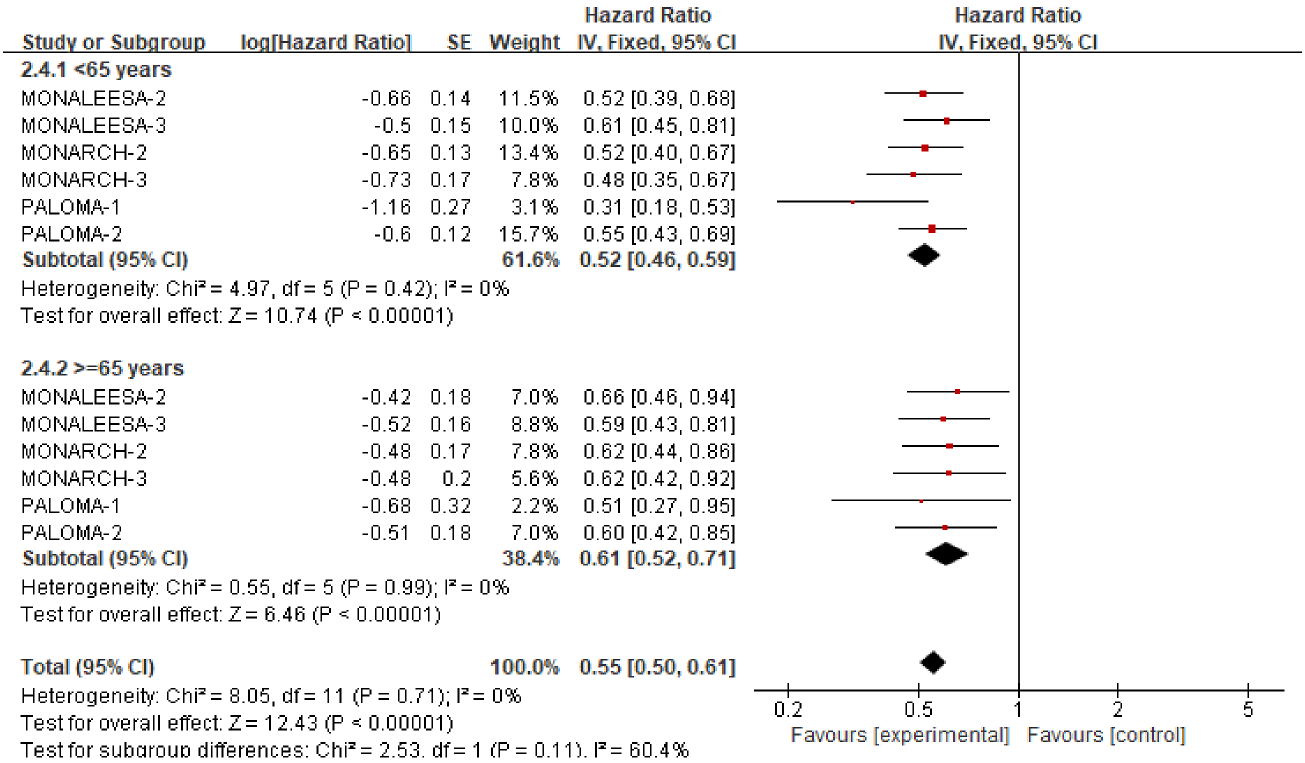

S1 Fig. Forest plot of hazard ratio for progression-free survival (PFS) in age.

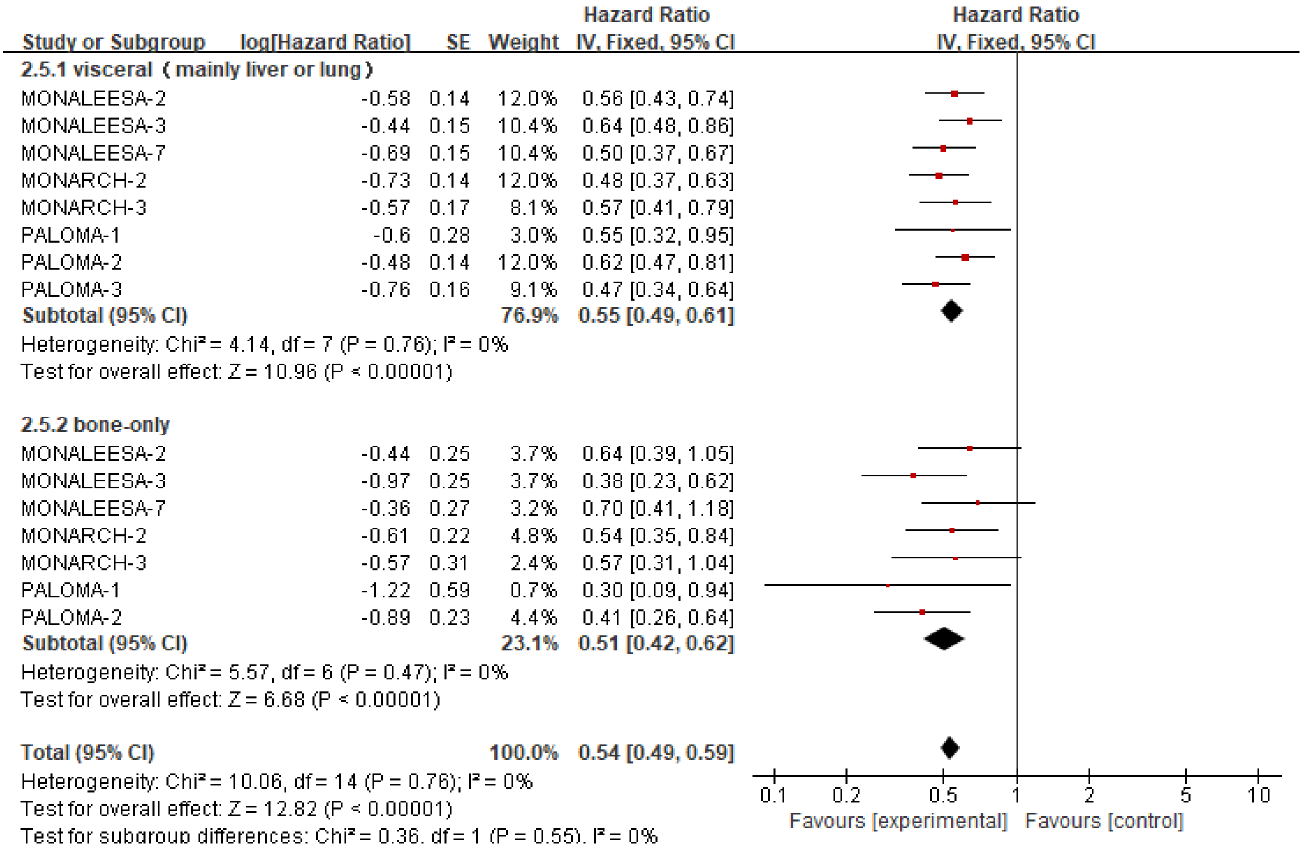

S2 Fig. Forest plot of hazard ratio for progression-free survival (PFS) in site of metastatic

disease.

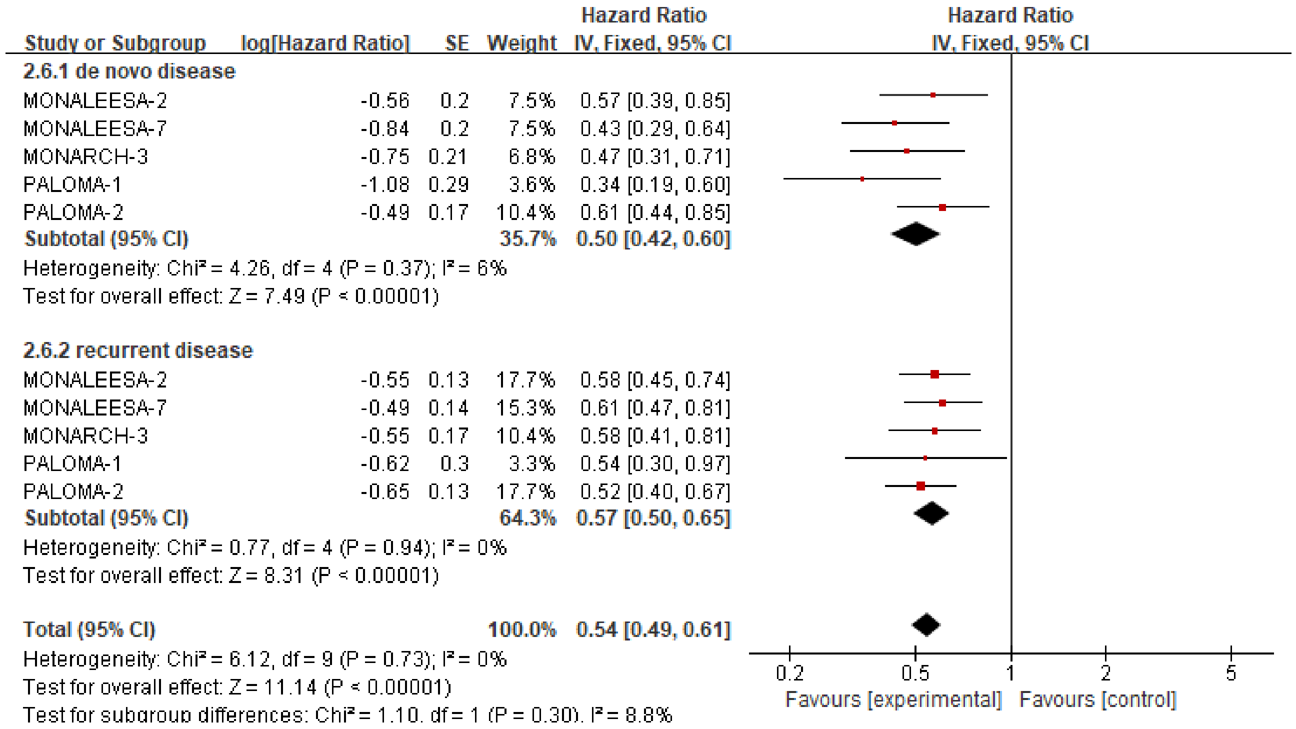

S3 Fig. Forest plot of hazard ratio for progression-free survival (PFS) in disease setting.

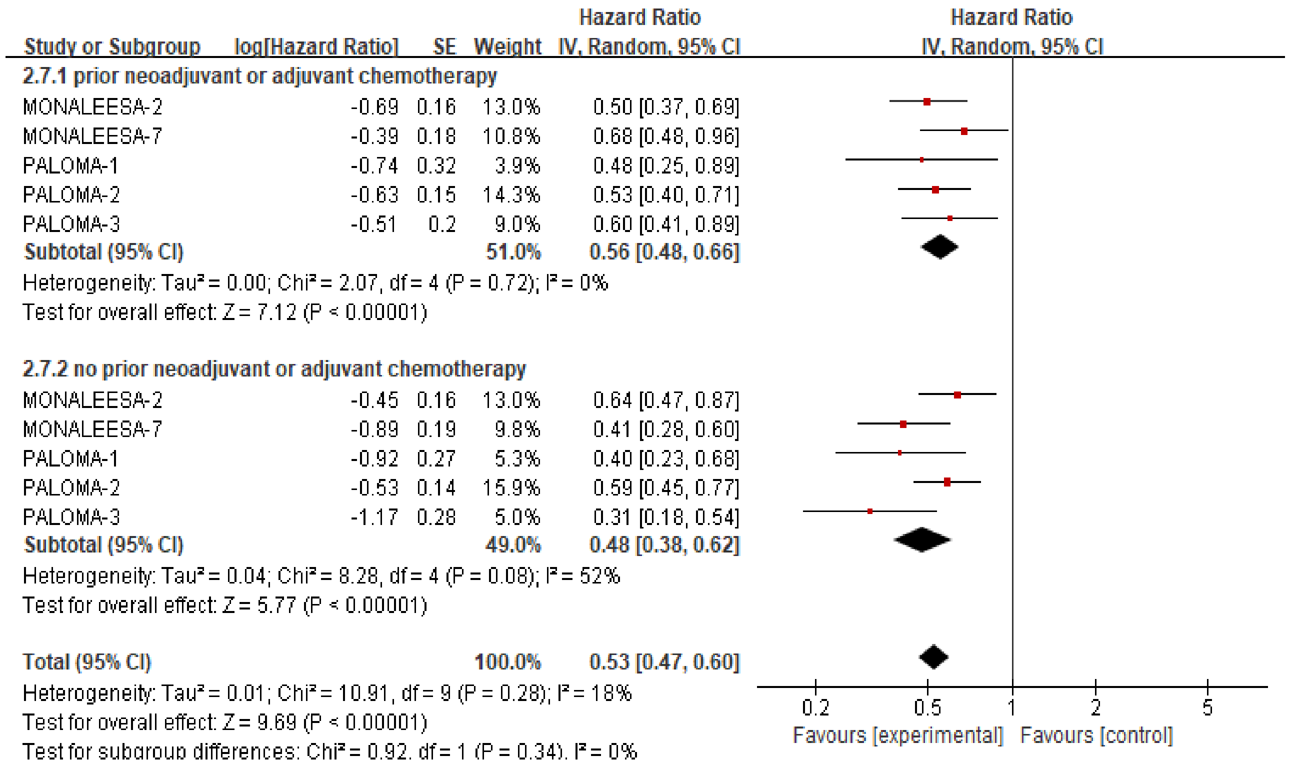

S4 Fig. Forest plot of hazard ratio for progression-free survival (PFS) in prior neoadjuvant or adjuvant chemotherapy.

Study or Subgroup	log[Hazard Ratio]	SE	Weight	Hazard Ratio IV, Fixed, 95% CI
**2.8.1 prior neoadjuvant or adjuvant endocrine therapy**				
MONALEESA-2	-0.69	0.14	15.1%	0.50 [0.38, 0.66]
MONALEESA-7	-0.48	0.18	9.1%	0.62 [0.43, 0.88]
MONARCH-3	-0.57	0.18	9.1%	0.57 [0.40, 0.80]
PALOMA-1	-0.78	0.37	2.2%	0.46 [0.22, 0.95]
PALOMA-2	-0.62	0.13	17.5%	0.54 [0.42, 0.69]
**Subtotal (95% CI)**			**52.9%**	**0.54 [0.47, 0.63]**
Heterogeneity: Chi² = 1.11, df = 4 (P = 0.89); I² = 0%				
Test for overall effect: Z = 8.22 (P < 0.00001)				
**2.8.2 no prior neoadjuvant or adjuvant endocrine therapy**				
MONALEESA-2	-0.43	0.17	10.2%	0.65 [0.47, 0.91]
MONALEESA-7	-0.65	0.16	11.5%	0.52 [0.38, 0.71]
MONARCH-3	-0.69	0.18	9.1%	0.50 [0.35, 0.71]
PALOMA-1	-0.92	0.25	4.7%	0.40 [0.24, 0.65]
PALOMA-2	-0.53	0.16	11.5%	0.59 [0.43, 0.81]
**Subtotal (95% CI)**			**47.1%**	**0.54 [0.47, 0.64]**
Heterogeneity: Chi² = 3.17, df = 4 (P = 0.53); I² = 0%				
Test for overall effect: Z = 7.68 (P < 0.00001)				
**Total (95% CI)**			**100.0%**	**0.54 [0.49, 0.60]**
Heterogeneity: Chi² = 4.28, df = 9 (P = 0.89); I² = 0%				
Test for overall effect: Z = 11.24 (P < 0.00001)				
Test for subgroup differences: Chi² = 0.00, df = 1 (P = 0.96), I² = 0%				

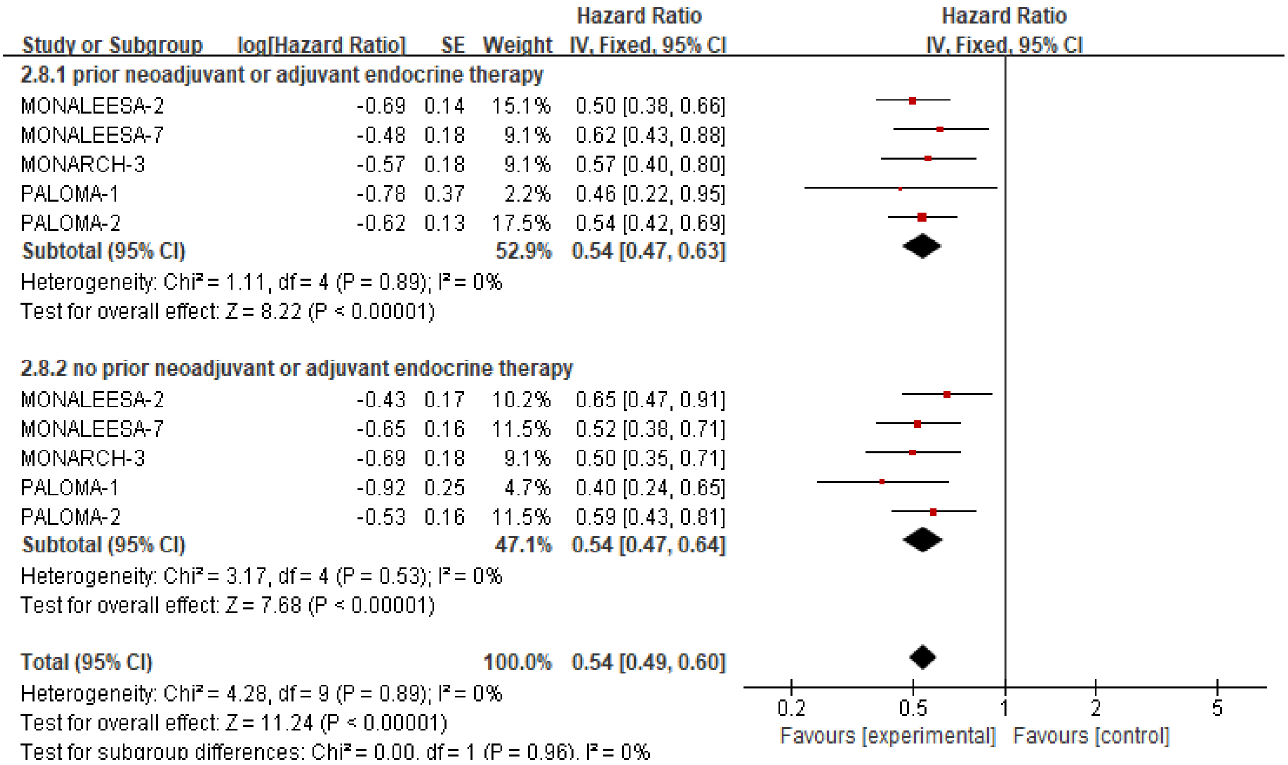

S5 Fig. Forest plot of hazard ratio for progression-free survival (PFS) in prior neoadjuvant or adjuvant endocrine therapy.

Study or Subgroup	log[Hazard Ratio]	SE	Weight	Hazard Ratio IV, Fixed, 95% CI
**2.9.1 ECOG 0**				
MONALEESA-2	-0.54	0.14	10.1%	0.58 [0.44, 0.77]
MONALEESA-3	-0.58	0.14	10.1%	0.56 [0.43, 0.74]
MONALEESA-7	-0.6	0.14	10.1%	0.55 [0.42, 0.72]
MONARCH-2	-0.72	0.14	10.1%	0.49 [0.37, 0.64]
MONARCH-3	-0.62	0.17	6.9%	0.54 [0.39, 0.75]
PALOMA-1	-0.83	0.29	2.4%	0.44 [0.25, 0.77]
PALOMA-2	-0.43	0.15	8.8%	0.65 [0.48, 0.87]
**Subtotal (95% CI)**			**58.4%**	**0.55 [0.49, 0.62]**
Heterogeneity: Chi² = 2.85, df = 6 (P = 0.83); I² = 0%				
Test for overall effect: Z = 10.18 (P < 0.00001)				
**2.9.2 ECOG >=1**				
MONALEESA-2	-0.61	0.18	6.1%	0.54 [0.38, 0.77]
MONALEESA-3	-0.46	0.17	6.9%	0.63 [0.45, 0.88]
MONALEESA-7	-0.69	0.22	4.1%	0.50 [0.33, 0.77]
MONARCH-2	-0.42	0.16	7.7%	0.66 [0.48, 0.90]
MONARCH-3	-0.64	0.21	4.5%	0.53 [0.35, 0.80]
PALOMA-1	-0.92	0.3	2.2%	0.40 [0.22, 0.72]
PALOMA-2	-0.67	0.14	10.1%	0.51 [0.39, 0.67]
**Subtotal (95% CI)**			**41.6%**	**0.55 [0.48, 0.63]**
Heterogeneity: Chi² = 3.52, df = 6 (P = 0.74); I² = 0%				
Test for overall effect: Z = 8.58 (P < 0.00001)				
**Total (95% CI)**			**100.0%**	**0.55 [0.51, 0.60]**
Heterogeneity: Chi² = 6.38, df = 13 (P = 0.93); I² = 0%				
Test for overall effect: Z = 13.32 (P < 0.00001)				
Test for subgroup differences: Chi² = 0.00, df = 1 (P = 0.99), I² = 0%				

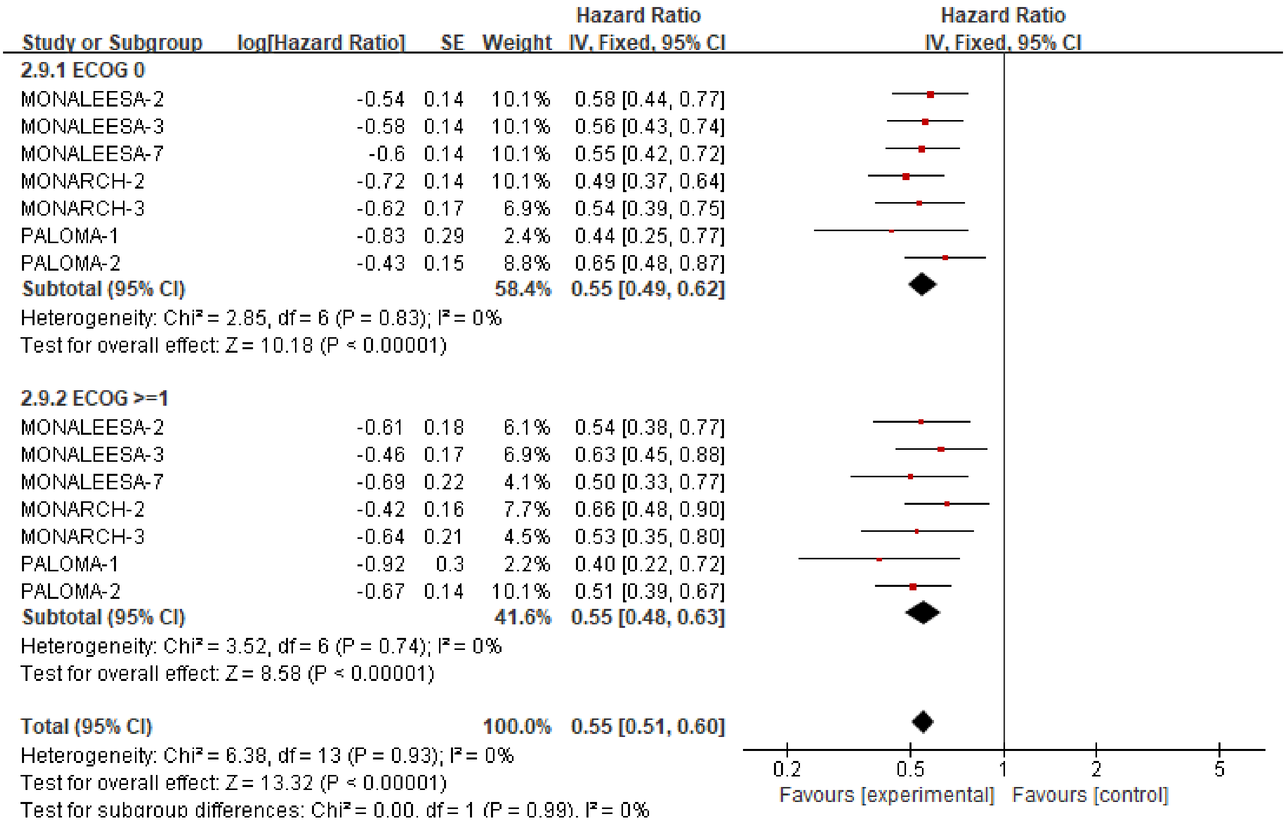

S6 Fig. Forest plot of hazard ratio for progression-free survival (PFS) in ECOG.

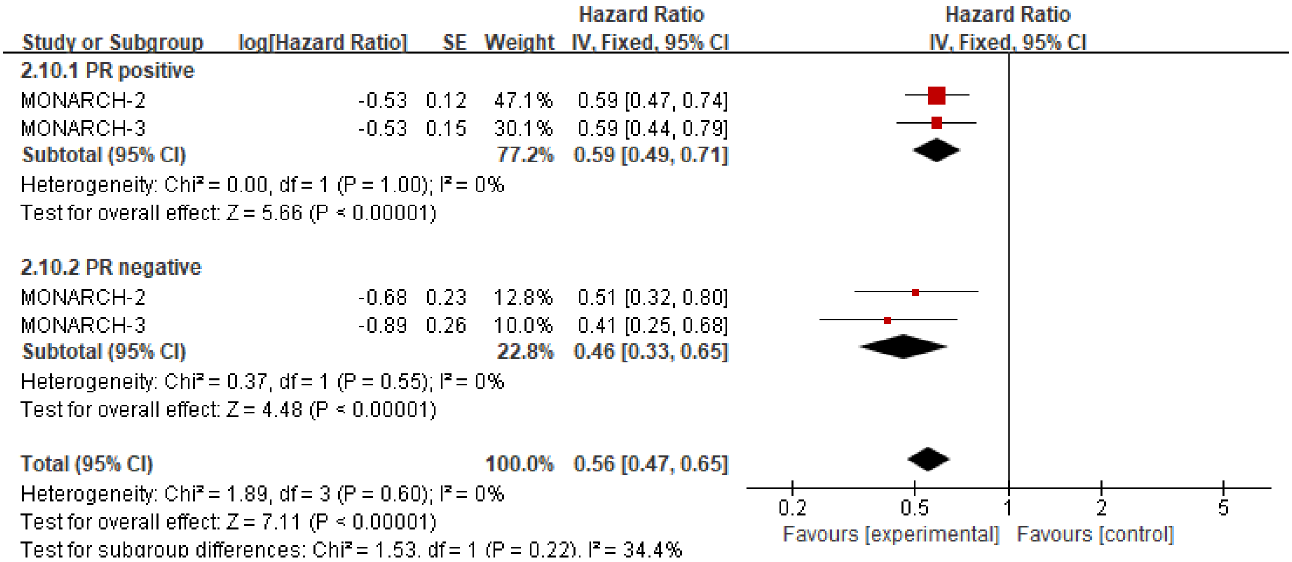

S7 Fig. Forest plot of hazard ratio for progression-free survival (PFS) in progesterone receptor status.

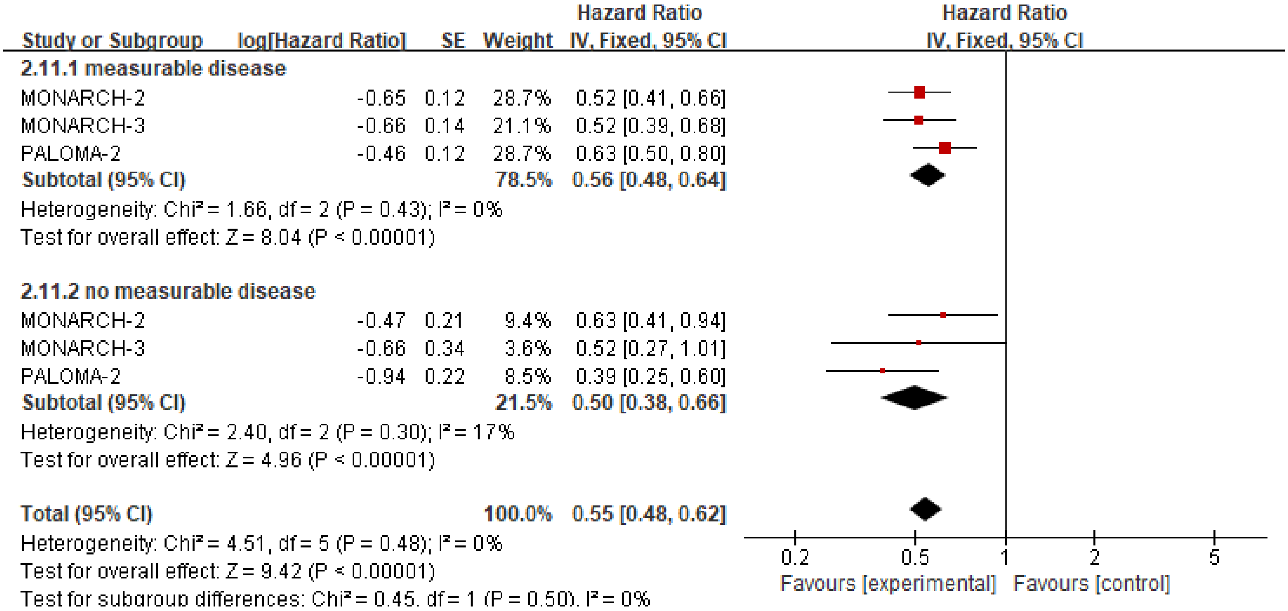

S8 Fig. Forest plot of hazard ratio for progression-free survival (PFS) in measurable disease.

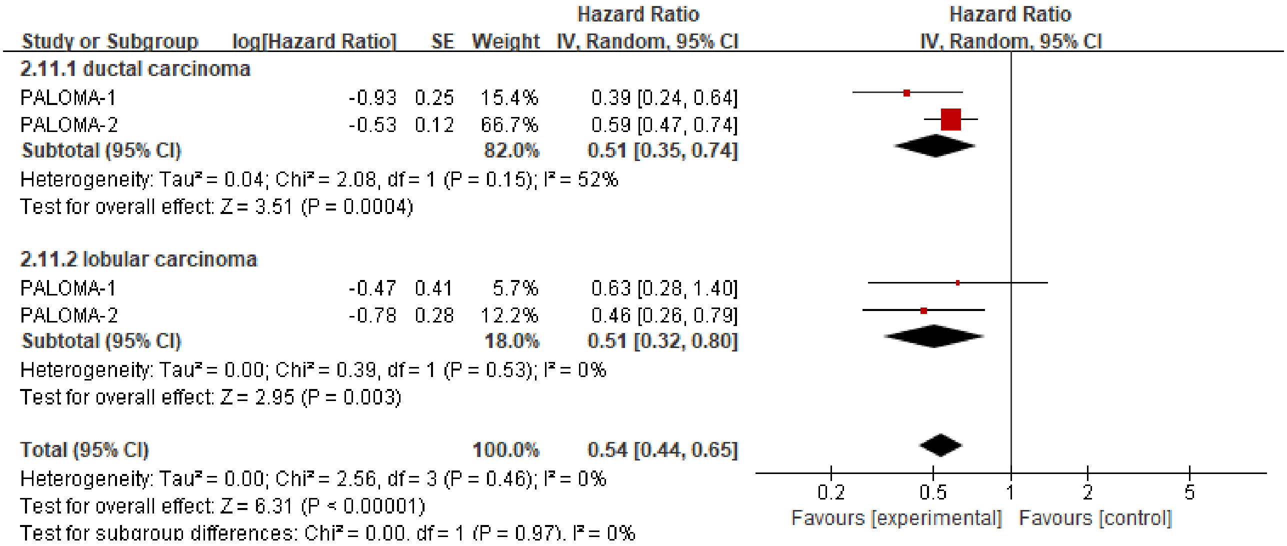

S9 Fig. Forest plot of hazard ratio for progression-free survival (PFS) in histopathological classification.

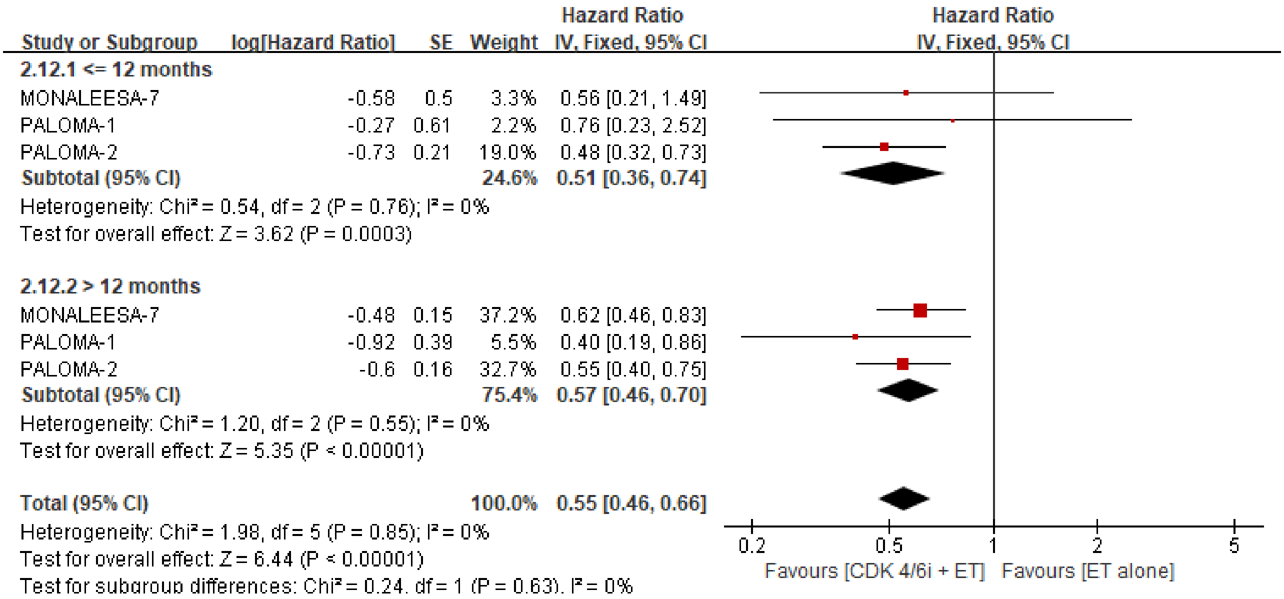

S10 Fig. Forest plot of hazard ratio for progression-free survival (PFS) in disease-free interval.

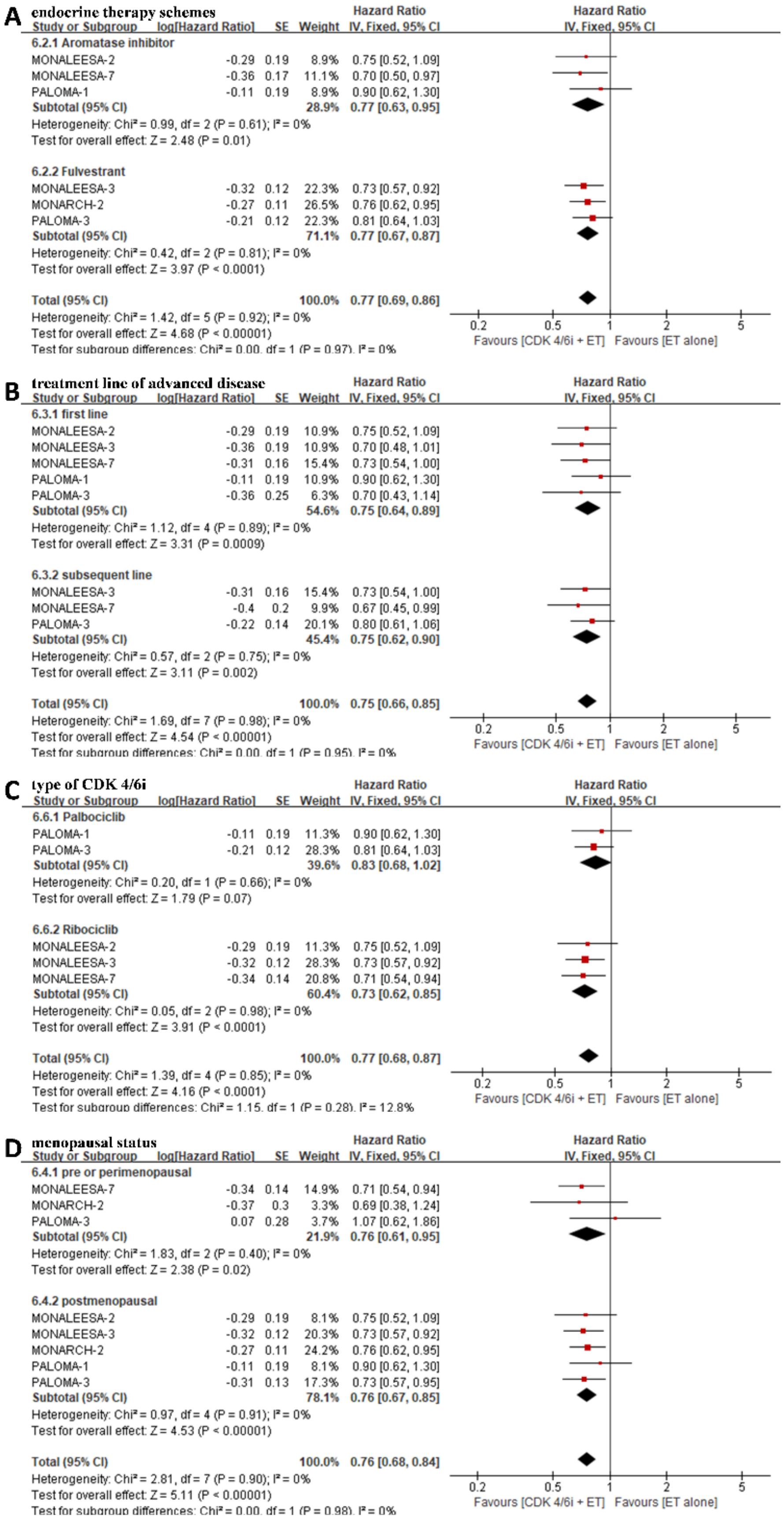

S11 Fig. Forest plot of hazard ratio for over survival (OS) in endocrine therapy schemes (A), treatment line of advanced disease (B), type of CDK4/6 inhibitors (C), menopausal status (D).

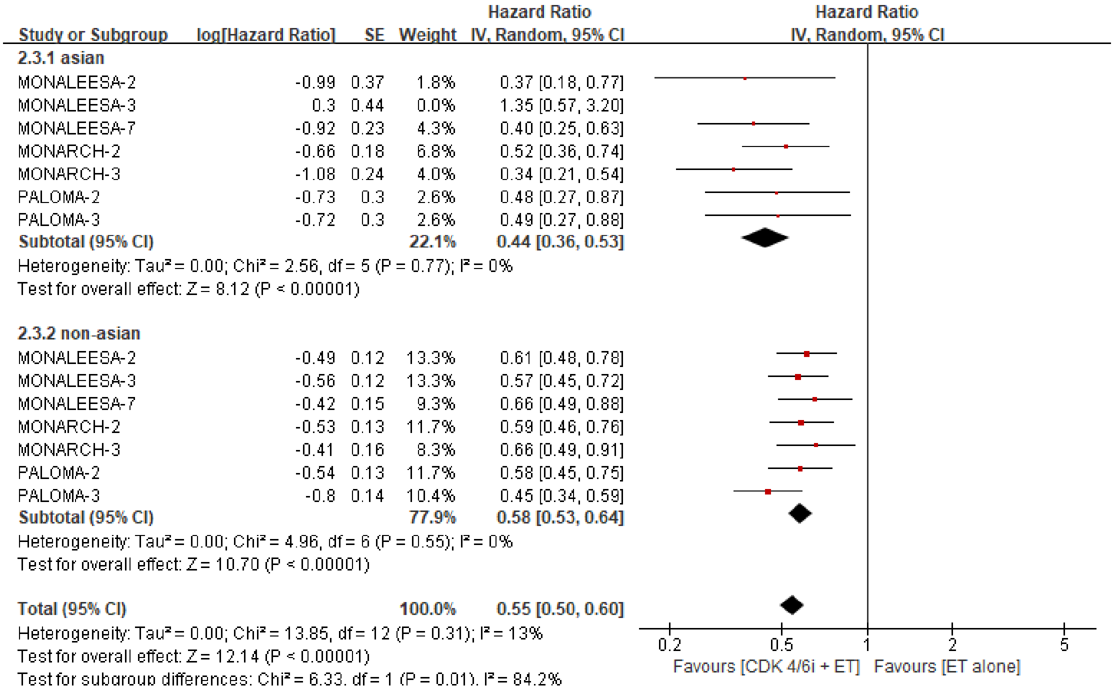

S12 Fig. The p-value of test for subgroup differences changed in race on progression-free survival (PFS) after excluded MONALEESA-3.
